# Case Report: Pulmonary hemorrhage as a rare cause of lung ultrasound A/B-profile

**DOI:** 10.12688/f1000research.19329.2

**Published:** 2019-08-08

**Authors:** Mark E. Haaksma, Esther J. Nossent, Paul Elbers, Pieter Roel Tuinman

**Affiliations:** 1 Research VUMC Intensive Care (REVIVE), VU University Medical Center, Amsterdam, Amsterdam, The Netherlands; 2 Amsterdam Medical Data Science (AMDS), VU University Medical Center, Amsterdam, Amsterdam, The Netherlands; 3 Amsterdam Cardiovascular Science (ACS), VU University Medical Center, Amsterdam, Amsterdam, The Netherlands; 4 Amsterdam Infection and Immunity Institute (AI&II), VU University Medical Center, Amsterdam, Amsterdam, The Netherlands; 5 Intensive Care, VU University Medical Center, Amsterdam, Amsterdam, The Netherlands; 6 Amsterdam Leiden Focused Intensive care Focused Echography (ALIFE), VU University Medical Center, Amsterdam, Amsterdam, The Netherlands; 7 Pulmonology, VU University Medical Center, Amsterdam, Amsterdam, The Netherlands

**Keywords:** Lung Ultrasound, BLUE-Protocol, Pulmonary Hemorrhage

## Abstract

When using lung ultrasound to determine the cause of acute respiratory failure, the BLUE protocol is often used. In a 65-year old patient, an A/B-profile was found, suggesting pneumonia, following the flowchart of this protocol. In this case, however, pulmonary hemorrhage confirmed by bronchoscopy was the final diagnosis. This case report outlines the importance of understanding the limitations of the BLUE protocol and that lung ultrasound findings should always be used in the context of the patient’s history and physical exam. In addition, pulmonary hemorrhage should be considered in patients with no clinical signs of pneumonia and/or presence of risk factors for lung bleeding as a rare cause of lung ultrasound A/B-profile.

## Introduction

Over the last few years, lung ultrasound has found routine use in critical care, even outperforming chest X-ray in detecting lung pathology ^1, 2^. Systematic approaches for its application are advised and have already been developed. For example, the BLUE protocol, developed by Lichtenstein *et al*., is often used to rapidly identify the cause of acute respiratory failure, with a claimed accuracy of 90.5% ^1^. It uses interpretation of artifacts visible in the presence of pleural and/or pulmonary pathology on three distinct places. These include the upper BLUE-point on the midclavicular line, the lower BLUE-point more caudally and lateral and the PLAPS-point (posterolateral alveolar and/or pleural syndrome point) on the dorsal side of the patient, continuing horizontally from the lower BLUE-point. The most frequently seen artifacts are lung-sliding and A-lines. The first being movement of the visceral pleura sliding against the parietal pleura and the second being a reverberation artifact of the pleural line. Their presence indicates unaffected lung surface. B-lines are vertical hyperechogenic artifacts and are linked to fluid buildup in lung tissue. The presence of more than two B-lines indicates interstitial syndrome. Combining these artifacts on every point and following the schema, the physician is led to five distinct diagnoses, which are cardiogenic pulmonary edema, pulmonary embolism, pneumothorax, obstructive disease or pneumonia. The latter being characterized by unilateral lung rockets resulting in an A/B-profile.

In the following article we present a case of pulmonary hemorrhage as a rare cause of lung ultrasound A/B-profile.

## Case presentation

**Figure 1.**
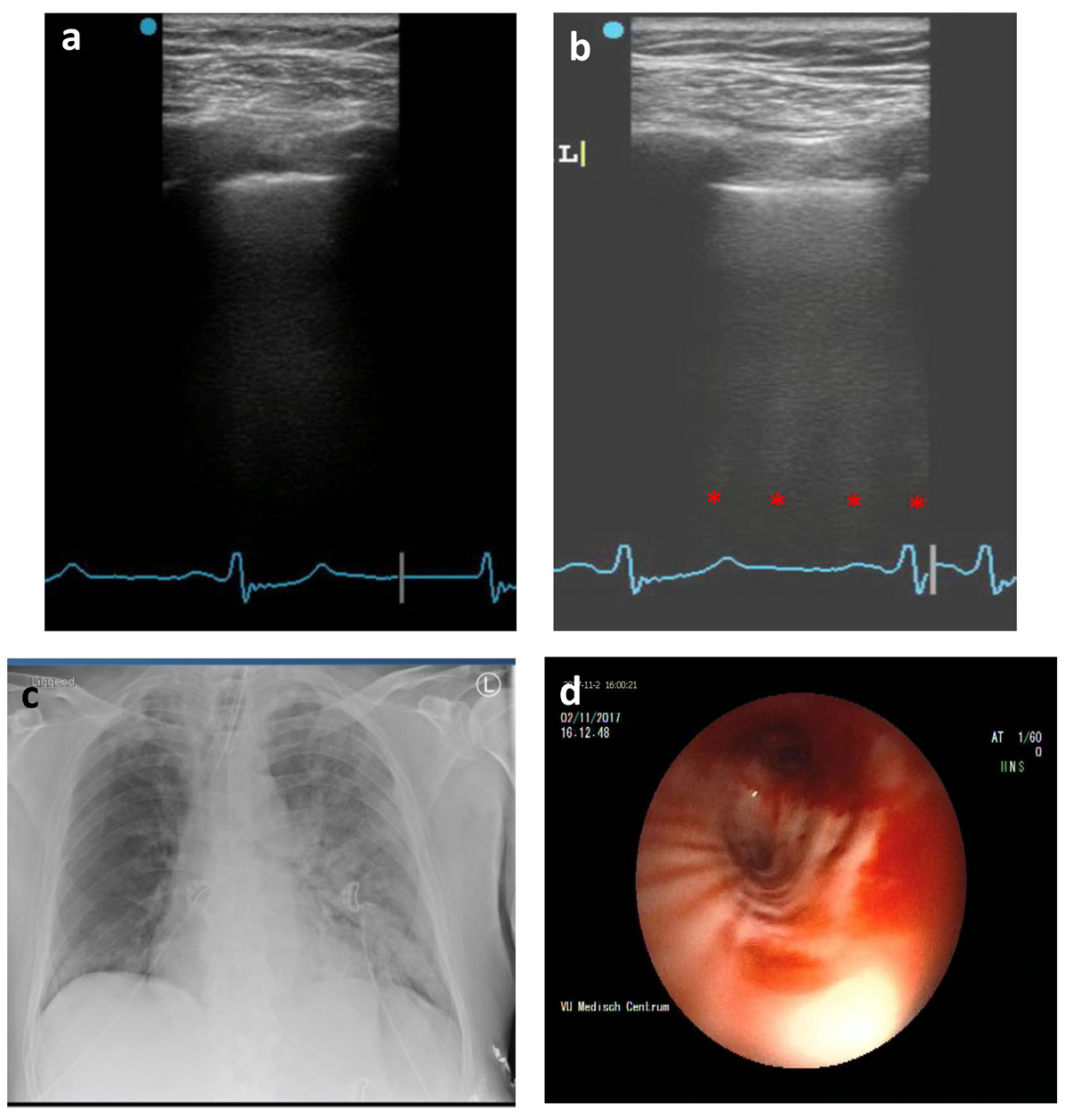
Images taken of the patient’s lungs. ( **a**) A-profile (right lung). ( **b**) B-profile with four distinguishable B-lines (left lung). Red dots, B-lines. ( **c**) Chest X-ray with marked consolidation in the left lung. ( **d**) Bronchoscopy of left main bronchus displaying bleeding from the upper lobe.

A 65-year old Caucasian male presented to the emergency ward with hemoptysis and respiratory distress. The patient’s history contained multiple pulmonary embolisms and subsequent chronic thromboembolic pulmonary hypertension, for which he was using acenocoumarol. Additionally, a non-specified form of pulmonary vasculitis was suspected, with concomitant treatment of prednisone. Bronchoscopy revealed pulmonary hemorrhage originating from the right pulmonary artery, which was coiled, and ICU admission followed. Two days after admission the patient was stable and extubated but showed signs of respiratory distress within the same day. Non-invasive ventilatory assistance failed and the patient was reintubated, while tranexamic acid (intravenous single dose, 1000 mg) was started as the cause was suspected to be a rebleed. Ultrasound examination according to the BLUE-protocol showed an A/B-profile ( Figure 1a, b) and chest X-ray showed left-sided consolidation ( Figure 1c). Subsequent bronchoscopy revealed a bleed originating from the left upper lobe ( Figure 1d) which was successfully coiled.

The following days were marked by respiratory instability and tracheal tube occlusion due to blood clots. One week after admission the patient developed atrial fibrillation, which was only temporarily relieved by cardiac resynchronization and amiodarone (300 mg intravenous loading dose in addition to 1200 mg/24 h intravenous for 1 day). This caused a further decline in cardiopulmonary function. A day later CT-angiography was performed and showed a fully occluded right bronchial artery and widespread occlusions in the left lung because of thrombosis. Due to the lack of additional therapeutic options, palliative care was started in line with the patient’s wishes, after which he passed away.

## Discussion

Lung ultrasound has been demonstrated to be of high value in patients with respiratory symptoms. The BLUE-protocol is probably one of the most well-used protocols in these patients due to its ease of use and high accuracy. However, with all the enthusiasm around it, it is of vital importance to be aware of its limitations.

The protocol was originally developed for and tested in an emergency room population, although it is now frequently used in ICU patients, relying on a 90% diagnostic accuracy ^1^. We don’t know, however, whether the same accuracy can be achieved in such a radically different patient population. ICU patients are frequently ventilated and could potentially be diagnosed with multiple pulmonary problems at the same time. In addition, pathology might be more subtle or different pathologies are present at the same time. To our knowledge, it is not clear how the BLUE-protocol performs in a setting with more than one underlying disease. Equally, the prevalence of included diagnoses differs between the ICU and the emergency room. This could potentially also influence its accuracy.

Furthermore, the BLUE-protocol relies on simplification of reality by categorizing the etiology of dyspnea into five groups. This is also demonstrated by our case report, in which A/B profile was not caused by pneumonia but pulmonary hemorrhage. In line with this and the findings of our case report, we want to highlight how important it is to interpret the artifacts in the context of the patient’s history and physical exam. AB profile would normally suggest pneumonia as the underlying cause, but in the absence of infectious symptoms and parameters this seems unlikely. Pairing the profile with the patient’s recent history of pulmonary hemorrhage, this diagnosis also fits the picture. While this is only one example, it is not unlikely that other scenarios in which the seen artifact could be paired with more than one diagnosis can present itself.

Aside from ambiguity of profiles, another problem is the lack of certain diagnoses, such as ARDS (adult respiratory distress syndrome). In the ICU it is a frequently encountered problem, but has currently no place in the BLUE-protocol. Including more complex problems like these or even more rare diagnoses could presumably also impact its accuracy.

Lastly, we do not know what the optimal approach is, given that different available protocols have not been compared. Assessing only three points per hemithorax might not be sufficient and perhaps more comprehensive imaging with more areas investigated are necessary ^3, 4^. The optimal balance of areas screened vs. time spent on an exam is yet to be determined. While expert are reviews available, strict protocols in regards to choice of probe and their settings during examination are not available ^5, 6^. This also might influence the quality of images and hence the interpretation of artifacts. In the future, we therefore hope to see more elaborate guidelines that encompass detailed information for image acquisition. Inclusion of more diagnoses perhaps with more advanced lung ultrasound modalities, such as Doppler or 3D imaging, would help to develop lung ultrasound to an even more sophisticated diagnostic tool.

## Conclusion

This case report outlines the importance of understanding the limitations of the BLUE-protocol and that lung ultrasound, like other imaging modalities, should always be used in the context of the patient’s history and physical exam. Also, pulmonary hemorrhage should be considered in patients with no clinical signs of pneumonia and/or presence of risk factors for lung bleeding as a rare cause of lung ultrasound A/B-profile.

## Data availability

No data are associated with this article.

## Consent

Written informed consent for publication of clinical details and clinical images was obtained from the patient’s family.
