# Supplementary material for: Case Report: Pulmonary hemorrhage as a rare cause of lung ultrasound A/B-profile
Source: F1000Res. 2019 Aug 8;8:788. Originally published 2019 Jun 5. [Version 2] doi: 10.12688/f1000research.19329.2 (PMC6720040; doi:10.12688/f1000research.19329.2)
Supplement: Peer review history

# Peer review: Reviewer response for version 2

**DOI:** 10.5256/f1000research.22077.r52233

**Published:** 2019-08-28

**Authors:** Belaid Bouhemad

**Affiliations:** 1 Service d'Anesthésie-Réanimation Chirurgicale, CHU de Dijon, Dijon, France

Ok for me. All asked modifications were done. Good work, nothing to add.

I confirm that I have read this submission and believe that I have an appropriate level of expertise to confirm that it is of an acceptable scientific standard.

# Peer review: Reviewer response for version 1

**DOI:** 10.5256/f1000research.21188.r49575

**Published:** 2019-06-27

**Authors:** Daniel A. Lichtenstein

**Affiliations:** 1 Medical Intensive Care Unit, Hospital Ambroise Paré, APHP, Paris-West University, Boulogne, France

Reviewing this interesting case report was a fine opportunity to make basic reminders on the BLUE-protocol. We hope this will allow the readers to better understand how to use it.

 We briefly correct minor details. The authors speak of *BLUE-1* and *BLUE-2*. Following the lung ultrasound nomenclature, they should speak of upper and lower BLUE-points ^1^. Four lines before Conclusions, minor typo ( *with* perhaps *with*).

 This being said, the good point is the illustration of a rare cause of dyspnea (pulmonary hemorrhage) seen through the BLUE-protocol (and generating an A/B-profile). This is important to know for future uses. We now have to comment numerous other points (focusing on the most important for shortening our analysis).

 The article begins softly by a simple case report of a rare situation, and then increasingly challenges several areas of the BLUE-protocol, not specifically related to the topic on focus (the non infectious A/B-profile). While respecting and understanding the approach of the authors, our review will explain again what is the BLUE-protocol - a precious reminder for those who did not read this article.

 This case report is presented as a limitation of the BLUE-protocol. It should, on the contrary, be understood as a perfect illustration of its spirit. May we remind that the BLUE-protocol is a *clinical* protocol. First of all (this is specified in its title), it includes the critical notion that the patient is in respiratory failure. If the BLUE-protocol was independent from this detail, we would all have severe asthma or exacerbated COPD, that is, the nude profile. Second, the BLUE-protocol is only the ultrasound part of a clinical approach to the patient. The BLUE-protocol is always performed after having received simple data of a simple history and a simple physical examination (see below for this case report).  

 Later, the authors consider that “the BLUE-protocol relies on [over] simplification”, with only five groups of diseases. May we remind that it has considered 97% of the patients (adults, seen in the ER, all admitted to the ICU)? We also remind that the majority of diagnostic mistakes (and dramas) include these frequent causes of dyspnea, this is why the BLUE-protocol focuses on the real, daily life. By the way, the 3% of other patients had countless causes (including this interesting case report). Creating a decision tree with all causes of dyspnea would generate a decision tree beyond any description in terms of complexity. Anyway, an answer to the concern of the authors can be consulted in our latest textbook, the E-BLUE-protocol, that is, an extended BLUE-protocol ^2^. They will find more developments of each profile by using tools from simple (echocardiography e.g.) to complex and invasive procedures.

 For each of the usual diseases, the reader benefits from various values of accuracies, which indicates that the BLUE-protocol is not designed for giving but suggesting (more or less highly) diagnoses.

 Therefore, we are here in a caricatural situation where the doctor has immediately detected a particular setting and is therefore invited to promptly use additional tests (as the authors rightly did in this case report). Here for instance, the history immediately points on a very particular situation (a long history of pulmonary embolism), and the physical examination immediately detects a major sign (hemoptysis) (and, we guess, absence of fever). The BLUE-protocol can of course be performed, but for building a diagnosis, such patients are immediately excluded from its thought process, and managed independently.

 This being said, we will comment on several points through the article:

1. Lung sliding and A-lines are “unaffected lung tissue”. We rather speak of unaffected lung *surface. *We remind that the A-profile (anterior bilateral lung sliding plus predominant A-lines) can be seen in pulmonary embolism (generating the A-DVT-profile), pure posterior pneumoniae (A-V-PLAPS-profile) and COPD and/or asthma (nude profile).
2. The authors consider that three points are too limited. Yet they cautiously use the word “perhaps”, and we invite them to test the BLUE-protocol with more points. We have done this comparison since three decades and did not find a significant change in the accuracies. We never found time for submitting our data, and strongly encourage the authors to making this comparison, and publish it.
3. B-lines are “linked” to fluid buildup. This is one of four possibilites only. *Stricto sensu, *the B-line indicates fluid surrounded by air (in a ratio of 1%-99% roughly). This fluid can be interstitial; it can be transudative in the case of hydrostatic pulmonary edema (B-profile usually), or exudative in the case of pneumonia/ARDS (B’-profile in many cases). The fluid can be intra-cellular; normal in the case of a simple lung fissure (isolated B-line), or pathologic on the case of chronic interstitial disease (usually lung rockets).
4. “The presence of more than two B-lines” is not sufficient. The diagnosis of interstitial syndrome is based, not on a number, but on a concentration of B-lines. The problem is (very simply) solved if you add “between two ribs”, that is a distance of roughly 2 cm, that is, 3 B-lines every 2 cm.
5. “Unilateral B-lines resulting in an A/B-profile”. Please replace “B-lines” by “lung rockets” and it will be correct. The B-line is not a disease, we almost all have B-lines. Reminder, more than two B-lines between 2 ribs are called lung rockets.
6. The authors write: “it is not clear how the BLUE-protocol performs in a setting with more than one underlying disease”. If they raise the issue of mixt diseases, these cases are integrated in the E-BLUE-protocol. In the native article, such patients were excluded for obvious methodological reasons (this affected roughly 1% of the patients). If a patient has two conditions potentially generating dyspnea, which gold standard will be able to determine the respective part of both diseases? It can be 50-50%, it can also be 51-49%, up to, potentially, 99-1%.
7. The authors write that ARDS has “currently no place in the BLUE-protocol”. This is wrong. The lecture of the native article shows that ARDS is considered as a pneumonia (severe, extensive, deadly, but just a pneumonia), which makes sense in the large majority of cases, again for simplifying a daily situation. The four profiles of pneumonia (B’, AB, C and AVPLAPS-profile) are found in the majority of cases of ARDS.  
8. The authors “don’t know whether the same accuracy can be achieved in such a radically different patient population” (emergency room versus ICU). First, some diagnoses can perfectly be achieved in ICU patients. A pneumothorax still is a pneumothorax. A major pleural effusion is still a pleural effusion. An increase of lung rockets (from septal rockets to glass rockets, e.g.) indicates a worsening (and many other examples). Yet the authors are partially right, these are two different groups. The answer is simple. The BLUE-protocol is reserved for the initial diagnosis (usually in the ER). For ICU patients, submitted to mechanical ventilation and severe diseases with fast changes, the CEURF (our academic teaching centre) has designed a specific approach, the Pink-protocol. These patients have no dyspnea and no cyanosis (hence this label), but they have no more lung function. The technique is more comprehensive that the BLUE-protocol (more points of investigation), and the interpretation is, obviously, more complex. Obstructive atelectases, e.g., much more frequent in this group, are integrated within the Pink-protocol. 
9. According to the authors, there is “no strict guidelines in regard to choice of probe”. We just recall the probe which we have used through all our publications on whole body ultrasound since our first success, submitted since 1991 ^3^. We use it for assessing abdominal disorders, deep venous thromboses, and all our procedures (including subclavian cannulation). We do not prevent the community to use classical probes, we just see the drawbacks of this approach: cost (buying probes), time (changing probes), infectious issues (cleaning probes - we are unable to avoid asepsis errors when using more than one probe) and last, a more complicated approach to the subtle science of lung ultrasound. Our probe is unique because of its logical ergonomy (allowing all accesses), its range allowing to detect both superficial and deep details (using the criteria 2 and 4 of the B-line), and its clear ability to visualise B-lines (using criteria 5 and 7) (Fig. 1).
10. In the Figure 1B, we do not see any B-line. Or, we just guess some, ill-defined, in the fog - and our 3-decade experience helped us a lot. How about novice users? The criterion N°5 of the B-line: “well-defined” (laser like), and the criterion N°7: “hyperechoic” (that is, white like the pleural line) are missing. This is a severe concern. Look our world (available since at least 1982), and please compare (Fig. 1). The result is a hindrance to an optimal development of lung ultrasound, and the need for complicated solutions. With a simple machine and probe, B-lines need no sophistication, no artificial intelligence, no automatic detection, no 3-D nor Doppler imaging. 

**Suggested solutions:**

 Please try to disactivate *all* filters of your modern machine, maybe you will succeed to have a convincing image. The manufacturer must not only sell you the unit, they must also find where are all those filter which, to our opinion, have no utility in critical ultrasound but just resulting in such images.

 The authors argue for Doppler and 3D imaging. Here, our answer is simple. Since three decades, we promise to use these sophisticated techniques as soon as we will feel limited by the simple and standardized approach provided by CEURF. This time has still not come. When we see the provided image quality, we understand why people develop sophisticated solutions. The physicians who use suitable equipment will, likely, not need these sophistication's. On purpose, we insist on this point.

 We take the advantage of this providential tribune for pointing out that the value of “100%” likely does not exist in medicine (apart from privileged exceptions, such as the lung point). In the native article, the A/B-profile was 100% specific to pneumonia. This beautiful performance was of course due to a weak number (12 of 300 studied patients had the A/B-profile). It is completely intuitive that multiplying the numbers will make appear exceptional situations. If we make a study of 1000 cases of AB-profile, rare diseases will be seen sometimes. We will see maybe 10 or 20 noninfectious causes, not a lot more we guess. Therefore, the specificity of the A/B-profile would become 99%, maybe 98%, not less. This is the basic statistical art.

 We are of course sorry about the outcome of this patient. Some academicians may argue that if a tool is used in an exceptional setting, makes anyway a diagnosis, but does not help the patient from a sad outcome, this tool was eventually of limited value here. We respect life too much for just thinking this.

**Conclusions:**

 This review was a nice opportunity for a pedagogic exercise: reminding what is the BLUE-protocol. If we were asked to summarize our review in a few words, we would write: “Please pilot the BLUE-protocol. Be a doctor... and it will give its best”. We congratulate the authors to use this attractive part of lung ultrasound, and we encourage them for any further investigations.

**Figure 1:** (Figure 2 ^4^ (middle image))

 B-lines, as they were described in the CEURF nomenclature with their seven criteria. Three are constant (comet-tail artifact, arising from the pleural line, moving in sync with lung sliding). Four are almost constant (long, well-defined, erasing A-lines, hyperechoic). Here, typical lung rockets. From a 1992 Hitachi 405 and its microconvex probe. 

 Note that in the figure under link, the terms *B7 and B3 lines *were long replaced by the much more speaking *septal rockets and glass rockets*, respectively.

I confirm that I have read this submission and believe that I have an appropriate level of expertise to confirm that it is of an acceptable scientific standard.

## References

[1] : The BLUE-points: three standardized points used in the BLUE-protocol for ultrasound assessment of the lung in acute respiratory failure. *Critical Ultrasound Journal*.2011;3(2) : 10.1007/s13089-011-0066-3 109-110 10.1007/s13089-011-0066-3

[2] : Lung Ultrasound in the Critically Ill. *Springer-Verlag International*.2016;

[3] : Intensive use of general ultrasound in the intensive care unit. *Intensive Care Medicine*.1993;19(6) : 10.1007/BF01694712 353-355 10.1007/BF01694712 8227728

[4] : A-lines and B-lines: lung ultrasound as a bedside tool for predicting pulmonary artery occlusion pressure in the critically ill. *Chest*.2009;136(4) : 10.1378/chest.09-0001 1014-1020 10.1378/chest.09-0001 19809049

# Response

**Published:** 2019-07-26

**Authors:** Mark Haaksma

**Affiliations:** Vumc, The Netherlands

Dear Dr. Lichtenstein,

Thank you for your time and expertise. We have adjusted the manuscript according to you feedback and would also like to respond to your comment. 

We would like to point out that this article was in no way meant to directly critique the BLUE-protocol. We ourselves are avid users of said protocol and ultrasound in general. The enthusiasm around it is, rightfully so, rapidly spreading. With it however, we have noticed that eagerness to quickly implement LUS in daily ICU practice comes with a lack of caution. Therefore, this article is meant as a reminder of certain caveats, which do not necessarily stem from the protocol but its implementation.

We agree that in this instance the cause for respiratory failure was relatively clear and that application of the BLUE protocol was not called for. However, we believe with this clear-cut situation we underline our points given in the discussion and above. Point being that the high accuracy of the protocol should not lead physicians to a misplaced sense of security in more dubious cases. This again, is more as consequence of its implementation and not the protocol itself. 

In regard to image and probes we agree that the presented microconvex probe probably has various benefits over other probes that are frequently used. However, exactly the fact that various probes are being used is of great importance. Physicians should, yet sometimes might not be, aware of this. In our daily practice, we also see that there are differences in image quality depending on probes used. 

While we agree that “simple” LUS is sufficient for B-line assessment, we still believe that in the spirit of scientific curiosity we are obliged to research whether arising modalities such as 3D and doppler imaging might contribute to it. Perhaps new possibilities will present itself in this regard.

Nevertheless, we want to thank you for your elaborate comment. It is without a doubt a good reminder and lesson of the BLUE-protocol for all readers.

# Peer review: Reviewer response for version 1

**DOI:** 10.5256/f1000research.21188.r49573

**Published:** 2019-06-21

**Authors:** Belaid Bouhemad

**Affiliations:** 1 Service d'Anesthésie-Réanimation Chirurgicale, CHU de Dijon, Dijon, France

Authors described limitations of the «BLUE protocol» about one case of hemoptysis. The authors should be commended for this task. The case report is well written, “English” seems to me very good. I have only 2 comments to add to the discussion part:

1. Blue protocol is not for ICU, we agree, and Lung US ICU protocols or guidelines are not available. But review written by experts are available. Please quote Bouhemad Anesthesiology, 2015 Feb;122(2):437-47 ^1^,  and Mojoli, Am J Respir Crit Care Med, 2019 Mar 15;199(6):701-714 ^2^. Moreover, in these papers, the issues of the needed “examinations points” (at least 6 X right/left) and probe are discussed.
2. In the conclusion, authors should precise that lung ultrasound "as all imaging technique should be considered in the patient’s….etc". 

I confirm that I have read this submission and believe that I have an appropriate level of expertise to confirm that it is of an acceptable scientific standard, however I have significant reservations, as outlined above.

## References

[1] : Ultrasound for. *Anesthesiology*.2015;122(2) : 10.1097/ALN.0000000000000558 437-47 10.1097/ALN.0000000000000558 25501898

[2] : Lung Ultrasound for Critically Ill Patients. *Am J Respir Crit Care Med*.2019;199(6) : 10.1164/rccm.201802-0236CI 701-714 10.1164/rccm.201802-0236CI 30372119

# Response

**Published:** 2019-07-26

**Authors:** Mark Haaksma

**Affiliations:** Vumc, The Netherlands

Dear Dr. Bouhemad,

Thank you for your time and expertise. We have adjusted the manuscript according to you feedback. The mentioned reviews are indeed an excellent addition to this article. We also agree that all imaging modalities have to be seen in the context of the patient.
